# Isolation and Characterization of Lytic *Pseudomonas aeruginosa* Bacteriophages Isolated from Sewage Samples from Tunisia

**DOI:** 10.3390/v14112339

**Published:** 2022-10-25

**Authors:** Ismahen Akremi, Maya Merabishvili, Mouna Jlidi, Adel Haj Brahim, Manel Ben Ali, Anis Karoui, Rob Lavigne, Jeroen Wagemans, Jean-Paul Pirnay, Mamdouh Ben Ali

**Affiliations:** 1 Laboratory of Microbial Biotechnology, Enzymatics and Biomolecules (LBMEB), Center of Biotechnology of Sfax (CBS), University of Sfax, Road of Sidi Mansour km 6, P.O. Box 1177, Sfax 3018, Tunisia; 2 Laboratory for Molecular and Cellular Technology, Queen Astrid Military Hospital, Bruynstraat 1, B-1120 Brussels, Belgium; 3 Astrum Biotech, Business Incubator, Center of Biotechnology of Sfax (CBS), University of Sfax, Road of Sidi Mansour km 6, P.O. Box 1177, Sfax 3018, Tunisia; 4 Agrovet, Street of Tunis km 1, Soliman 8020, Tunisia; 5 Laboratory of Gene Technology, Department of Biosystems, KU Leuven, Kasteelpark Arenberg 21-Box 2462, B-3001 Leuven, Belgium

**Keywords:** bacteriophages, *Pseudomonas aeruginosa*, multidrug resistance, phage therapy

## Abstract

Bacteriophages could be a useful adjunct to antibiotics for the treatment of multidrug-resistant *Pseudomonas aeruginosa* infections. In this study, lytic *P. aeruginosa* myoviruses PsCh, PsIn, Ps25, and Ps12on-D were isolated from Tunisian sewage samples. Phage Ps12on-D displayed an adsorption time of ~10 min, a short latency period (~10 min), and a large burst size (~115 PFU per infected cell) under standard growth conditions. All phages were active at broad temperature (4 °C to 50 °C) and pH (3.0 to 11.0) ranges and were able to lyse a wide variety of *P. aeruginosa* strains isolated from clinical and environmental samples worldwide. Illumina sequencing revealed double-stranded DNA genomes ranging from 87,887 and 92,710 bp with high sequence identity to *Pseudomonas* phage PAK_P1. All four phages based on sequence analysis were assigned to the *Pakpunavirus* genus. The presented characterization and preclinical assessment are part of an effort to establish phage therapy treatment as an alternative strategy for the management of multidrug-resistant *P. aeruginosa* infections in Tunisia.

## 1. Introduction

Antibiotics are considered to be one of the greatest discoveries in modern medicine, leading to a substantial reduction in human morbidity and mortality [1]. However, their applicability is currently hampered by the rapid increase in bacterial resistance [1]. Several studies suggest that a strong link exists between antibiotic consumption and the emergence of antibiotic resistance [2]. In the era of antimicrobial resistance, *Pseudomonas aeruginosa* represents one of the most concerning bacterial pathogens involved in antibiotic-resistant infections, together with the five other representatives of the ‘ESKAPE’ group of pathogens: *Enterococcus faecium, Staphylococcus aureus, Klebsiella pneumoniae, Acinetobacter baumannii,* and *Enterobacter* spp. [3]. Worldwide, nosocomial infections caused by multidrug-resistant (MDR) ESKAPE pathogens are associated with the highest risk of death and enormous treatment costs [4]. 

According to WHO guidelines, carbapenem-resistant *P. aeruginosa* is a priority 1 (critical) bacterial pathogen for which the development of new antibiotics is urgently needed [5]. 

Antibiotic-resistant *P. aeruginosa* is causing life-threatening infections in individuals with compromised immune systems, including burn wound patients. This pathogen is the most common causative agent in chronic respiratory infections, as well as the leading cause of mortality in cystic fibrosis patients [4,5,6]. The severity of *P. aeruginosa* infections is the result of increased virulence and a high level of antibiotic resistance, both natural and acquired [7,8]. The pathogenicity of *P. aeruginosa* is attributed to the production of a large arsenal of virulence factors (membrane and extracellular) acting at different levels during infection and allowing the bacterium to survive both in different hosts and in the environment [9]. Moreover, they play an important role in tissue invasion by the bacterium [9]. In addition, *P. aeruginosa* is characterized by the plasticity of its genome, facilitating a straightforward and rapid acquisition of very diverse antimicrobial resistance mechanisms [8,10]. 

In Tunisia, the growing number of MDR strains has become a particularly serious health concern, contributing to high rates of mortality and morbidity. In this context, *P. aeruginosa* is one of the most problematic bacteria associated with nosocomial infections in intensive care units (ICUs) in Tunisia [11], especially since the usual therapeutic options, such as the use of tetracycline, are increasingly failing. A study carried out in various ICUs in Tunisia from 2009 to 2014 showed that *P. aeruginosa* caused 22.86% of all nosocomial infections [12]. The increasing frequency of imipenem-resistant *P. aeruginosa* isolates in Tunisia is becoming a serious public health concern. In a study performed by Krir et al. [13], *P. aeruginosa* strains isolated between 2012 and 2018 showed high percentages of resistance to beta-lactamases (77.1% of isolates) and imipenem (63.2%), which were considerably higher than those reported in other studies [14,15]. 

The continuing increase in antibiotic resistance of clinically relevant *P. aeruginosa* strains worldwide and a shortage of new antimicrobials in the drug development and marketing pipeline have created an urgent need to explore alternative strategies such as bacteriophage (phage) therapy. This therapy is increasingly put forward as a promising additional tool in the fight against MDR (*P. aeruginosa*-mediated) infections [16].

The concept of using phages to treat bacterial infections, called phage therapy, belongs to Félix d’Hérelle, one of the co-discoverers of phages, and dates back to the beginning of the 20th century. The discovery of bacteriophages active against the *Pseudomonas* genus dates back to the middle of the 20th century, and recently interest in them grew due to *P. aeruginosa*’s prevalent role in nosocomial infections and high levels of antibiotic resistance [17,18]. Several studies have shown the potential of phage applications in the treatment of *P. aeruginosa* infections, and particularly those caused by MDR strains [18,19]. Today, while sporadically applied in parts of the Western world, phage therapy is practiced on a routine basis in the Eliava Institute in Tbilisi (Georgia), a research center dedicated (since 1922) to the production and application of phage therapeutics [20]. To date, based on the outcome of several clinical trials and case reports, we can claim that phage therapy has yielded promising results in treatment of various kind infections caused by *P. aeruginosa* such as leg ulcers [21], burns [22,23], and ear infections [24]. In the PhagoBurn study [23], 25 patients were recruited and randomly assigned to receive treatment with phage cocktail PP1131 (n = 12) or standard of care (n = 13). The highest bacterial burden was found in eight patients from the two groups (six of them from the PP1131 group). The PP1131 overall titer decreased after manufacturing, and participants were given a lower concentration of phages than expected (1 × 10^2^ PFU/mL per daily dose). In the PP1131 group, three of the analyzable participants experienced adverse events, compared to seven in the standard-of-care group. In the scope of the PhagoBurn clinical trial, a salvage therapy treatment was performed in November 2015 on the patient with severe burns covering 80% of his body surface and infected with MDR *P. aeruginosa* [25]. Survival probability was less than 2%, and as a last-resort treatment anti-*P. aeruginosa* phage cocktail (PP1131) was applied. During the treatment (7 days), phage multiplication was monitored compared to bacterial growth. Although the initial concentration was low, in the range of 10^1^ 10 ^2^ PFU/mL, an intense phage multiplication occurred within 2 days of the treatment’s start, reaching 10^10^ PFU/mL as the bacterial concentration on the burnt wound decreased from 10^5^ to 10^1^ on the date of death [25]. In the study of the Eliava phage therapy center, Zaldastanishvili et al. [26] demonstrated that two patients who had a lower respiratory tract infection colonized with *P. aeruginosa* were successfully treated with phages without any adverse effects.

Phage therapy has been proven to be effective in treating *P. aeruginosa* infections in both humans and animals, according to a number of case studies and small clinical and preclinical trials [24,27,28,29].

In this study, we describe the isolation and biological characterization of four strictly lytic *P. aeruginosa* phages from Tunisia. We studied the whole-genome sequence of the four phages and demonstrated their lytic activity against MDR *P. aeruginosa* clinical isolates, in view of their inclusion in a repertoire of potential antipseudomonal therapeutic phages. A large part of the study was performed in Belgian phage research centers with collaboration of our Belgian colleagues. 

## 2. Materials and Methods

### 2.1. Bacterial Strains and Culture Conditions

The bacterial strains used in this study are listed in Supplementary Table S1. *P. aeruginosa* strain PaTun is a clinical isolate obtained from the Hospital Hedi Shaker in Sfax, Tunisia, and was used as a host strain for phage isolation. A collection of 140 clinical and environmental *P. aeruginosa* isolates from the collection of the Queen Astrid Military Hospital (Brussels, Belgium) [30] was also used (Supplementary Table S1). All strains were grown in Lysogeny Broth (LB, Becton Dickinson, Erembodegem, Belgium) medium at 37 °C aerobically.

### 2.2. Isolation and Propagation of Bacteriophages

*P. aeruginosa* strain PaTun was used as a phage host for the isolation of phages from four wastewater samples from different locations in Sfax, Tunisia, and more specifically from the Chahia Company, the Spiga Company, Sfax Airport, and the Sidi Salem Power Station and were isolated in the Astrum Biotech Laboratory. Water samples from these sites were filtered using 0.22 μm filters (Millipore, USA) to remove debris and bacteria. A 100 µl aliquot of the filtrate was added to 5 mL of *P. aeruginosa* culture in early log-phase and incubated at 37 °C for 24 h with constant shaking at 150 rpm to enrich for present phages. The culture was then centrifuged at 10,000× *g* for 20 min at 4 °C. Bacteriophages in the supernatant were tested for plaque formation using the standard double-agar layer method [31]. Single isolated plaques were picked to start a second round of amplification. The purification passages were repeated at least five times to ensure purity of the phage. To propagate these phages, the double-agar overlay method [22] was used with some modifications. In particular, lysates received after plate propagation on *P. aeruginosa* PaTun were centrifuged for 20 min at 6000× *g* and filtered through 0.45 μm filters and subsequently through 0.22 μm filters either using syringe filters or filtration unit systems. The phage suspension was further purified and concentrated by high-speed centrifugation at 35,000× *g* for 1.5 h and the obtained phage pellet was diluted in DPBS (Lonza, Belgium) of ten times less volume compared to the initial suspension. The received high-titer phage suspensions were stored at 4 °C.

### 2.3. Extraction, Sequencing, Annotation, and Taxonomic Assessment of the Phages and Bacterial Genomes

To digest the exogenous DNA and RNA, the purified phage samples were treated for 1 h at 37 °C with DNase I (Invitrogen) and RNase A (Invitrogen). Proteinase K (Tiangen Biotech) was then added to the preparation for 15 min at 55 °C. The phage genomic DNA was isolated further using the PureLinkTM Viral RNA/DNA Mini Kit. The Bacterial DNA Kit was used to extract the DNA from the *P. aeruginosa* PaTun strain (DNeasy UltraClean 96 Microbial Kit, Qiagen). The phage genomes and the PaTun bacterial genome were sequenced on an Illumina MiniSeq NGS platform (Illumina, San Diego, CA, USA). The Nextera Flex DNA library kit was used to prepare the library. Illumina reads were trimmed with Trimmomatic (v. 0.36.5) and assembled with Unicycler (v. 0.4.8.0) after sequencing [32,33]. Bandage was used to evaluate the quality of the contigs (v. 0.8.1). The PATRICbrc server (v. 3.6.2) was used to annotate genomes [34,35]. BLASTp [36] was used to manually curate the automated annotation. Alignments were made using the MUSCLE algorithm [37]. In addition, VIRIDIC [38] was used to create a heatmap that combined intergenomic similarity values with genome length and aligned genome fraction information. tRNAscan-SE 1.21 [39] was used to look for genes that code for tRNAs. The VirulenceFinder [40] was used to identify the virulence gene in phage genomes of interest, and by examining the input proteome, genome, or raw data provided by the user, the PathogenFind web service [41] has been described as being capable of predicting bacterial pathogenesis. Finally, the prophage regions were identified in the *P. aeruginosa* PaTun genome using the PHASTER (Phage Search Tool Enhanced Release) online tool, which scores putative phage regions [42]. PHASTER assesses the completeness of predicted phage-related regions based on the number of known genes/proteins found in the bacterial prophage region: intact (>90%), questionable (90–60%), and incomplete (60%) regions.

### 2.4. Host Range Analysis

The host range of the phages was determined using 140 clinical and environmental isolates of *P. aeruginosa* isolated from different sources and countries, using a dilution spotting assay [43]. Briefly, 100 μL of bacterial cultures, conferring 10^8^ colony forming units (CFU)/mL, was mixed with 4 mL of molten semi-solid LB broth 0.6% agar at 45 °C and overlaid on LB agar plates. Then, 10 μL of phage suspension in a titer of 10^8^, 10^6^, and 10^4^ plaque-forming units (PFU)/mL was applied onto the bacterial lawn and allowed to air dry. Following overnight incubation at 37 °C, plates were examined for lysis zone/plaque formation to define bacterial susceptibility to the phages. The obtained lysis zones were categorized as confluent lysis (CL), semi-confluent lysis (SCL), opaque lysis (OL), separate plaques (SP), and no activity (-). The results were interpreted as follows: when separate plaques were observed, phages were considered capable of adsorbing/infecting the strain; when only lysis zones were observed at the two highest tested concentrations, it was interpreted as killing from without. A potential bacteriocin effect could be excluded, as the host strain genome does not encode any of these. 

### 2.5. Thermal Stability and pH Sensitivity

For thermal stability test, phages were added to LB, and the mixtures were incubated at various temperatures: 4 °C, 25 °C, 37 °C, 50 °C, 60 °C, and 70 °C for 1 h. After incubation, the titer of each phage sample was determined by the double-agar layer method [31]. To investigate the effect of pH on phage infection, each phage (PsIn, PsCh, Ps25, and Ps12) at a concentration of 10^8^ PFU/mL was mixed with a series of phage buffers with different pH values (pH 3, 5, 7, 9, and 11), and the titer of phages was measured after incubation at 37 °C for 2 h. 

### 2.6. Phage Adsorption Assay 

*P. aeruginosa* CN573 was infected in LB at a multiplicity of infection of 0.01 to determine the kinetics of phage Ps12on-D, PsCh, PsIn, and Ps25 adsorption. Samples from the bacteria–phage mixture were taken at 1 min intervals, and titers of free phage particles were determined by the double-agar layer method [25] after removal of the bacterial cells. This was obtained by the addition of 2.0 % *v*/*v* chloroform to each sample and subsequent incubation for 1 h at 4 °C. The percentage of non-adsorbed phages was calculated at 0, 3, 5, 10, 15, 20, and 30 min. Adsorption curves were generated, plotting the ratio of unabsorbed phages to initial phage numbers at different time points. Adsorption rate constant was calculated according to Rombouts et al. [44] using the following equation:

K=2.3/(B) × Log(P_0_/P) (ml/min)

in which P_0_ = phage assay at zero-time, P = phage not adsorbed at time t min, (B) = concentration of bacteria as number of cells/mL, K = velocity constant with dimensions mL /min. 

### 2.7. One-Step Growth Curve 

The one-step growth experiment was carried out with minor modifications as previously described [45]. In brief, strain *P. aeruginosa* CN573 was grown in LB to the concentration of 1 × 10^9^ CFU/mL. The phage Ps12-on-D was then added to the CN573 culture at an MOI of 0.01 (equivalent to 1 × 10^7^ PFU/mL) and allowed to adsorb for 10 min at 37 °C. The mixture was centrifuged for 1 min at 14,000× *g* to remove non-adsorbed phages. The pellet of infected cells was resuspended in 10 mL of growth medium after being washed twice with fresh LB, and the culture was incubated at 37 °C for 60 min. The free phage count was determined by titration using the double-agar overlay method after samples were taken at 3, 5, 10, 20, 30, 40, 50, and 60 min. The latent period was calculated after the adsorption process was completed (>80%), and it is the time between infection and phage production. By dividing the phage titers at the plateau phase by the initial number of infectious bacterial cells throughout the latency period, the burst size was calculated.

### 2.8. Accession Numbers

The genome sequences of phage vB_Paer_Ps12on-D, vB_Paer_Ps25, vB_Paer_PsCh, and vB_Paer_PsIn were deposited in GenBank under the accession numbers OM870967, OM870968, OM870969, and OM870970, respectively.

## 3. Results and Discussion

### 3.1. Isolation and Selection of the Phages

Sixteen phage clones infecting *P. aeruginosa* PaTun were isolated from the wastewater from Tunisia. Four phages, vB_Paer_Ps12on-D, vB_Paer_PsCh, vB_Paer_PsIn, and vB_Paer_Ps25, were further selected to estimate their therapeutic potential, based on preliminary screening on eleven strains of *P. aeruginosa* representing different genotypes (Supplementary Table S1). The selected phages were isolated from different locations, and they showed differences in host range and plaque morphology. On double-layer agar plates, Ps12on-D, Ps25, and PsIn produced clear plaques of approximately 3 ± 0.8 mm in diameter, whereas phage PsCh produced bigger plaques of approximately 6 ± 1.0 mm. The plaques from all four phages demonstrated halos around a clear center on a lawn of the host bacterial strain. To obtain insight on identity and difference between the selected phages, they were analyzed using whole-genome sequencing.

### 3.2. Genomic Identification of the PsIn, PsCh, Ps12on-D, and Ps25 Phages

The genome sequence of the four selected phages was determined using Illumina sequencing, yielding between 42,372 and 48,628 raw reads with an average length of 148 bp. Assembly resulted in single contigs of 88,705, 92,710, 92,515, and 87,887 bp in length for phages Ps12on-D, PsCh, PsIn, and Ps25, respectively, with an average GC content of 49.21%.

The genome of all four phages showed high sequence identity (up to 97.43%) to the genome of *Pseudomonas* phages PAK_P1 (KC862297.1), PaP1 (HQ832595.1), YS35 (MF974178.1), and JG004 (GU988610.2) with an average of 95% coverage. These similar phages were isolated from different locations around the world, spanning Asia, Europe, and Africa, consistent with previous observations. Indeed, while local diversity can be high, global diversity is rather limited and similar phages can be found across the globe [46]. These phages also represent a group of phages with high therapeutic potential [47,48].

We calculated the intergenomic distance between the related phages using VIRIDIC [49], resulting in a maximum similarity between our phages and other known sequences of 94% (Supplementary Figure S1). Currently, for bacterial and archaeal viruses, the main species demarcation criterion is set at 95% genome sequence identity [50]. At the same time, if the sequence similarity between two phages is greater than 70%, they are considered to belong to the same genus. The four selected phages can therefore be considered as isolates from one new species in the *Pakpunavirus* genus, displaying a myovirus morphotype. 

**Figure 1:**
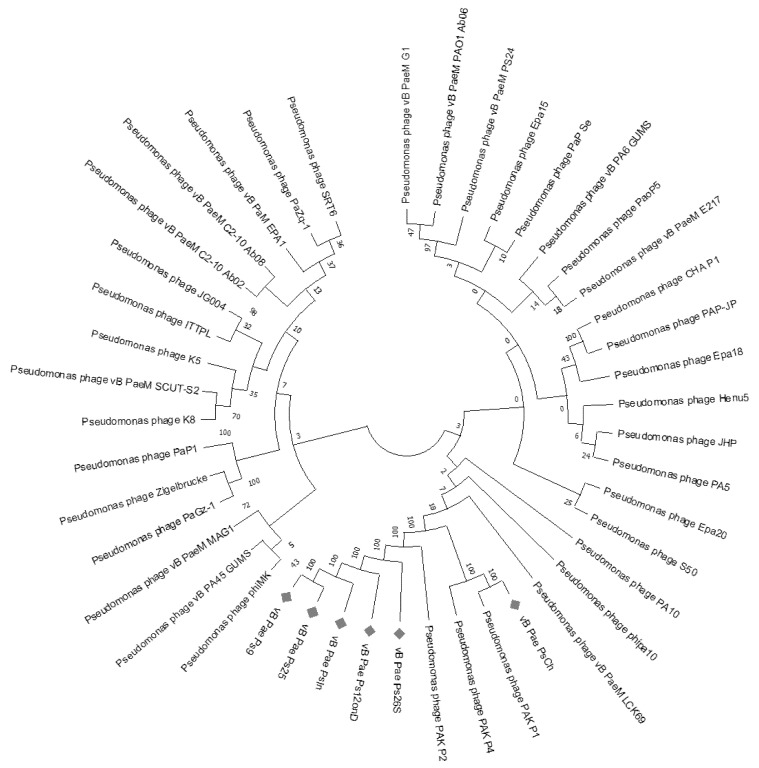
Phylogenetic tree based on whole-genome sequence comparisons of six selected phages, generated with MEGA X, version 10.2.4’s neighbor-joining method. The evolutionary distances were calculated using MEGA X’s Maximum Composite Likelihood method [51]. The percentage of replicate trees where the associated taxa clustered together during the bootstrap test is represented by the numbers below the branches (1000 replicates). The best tree is depicted to scale, with branch lengths representing the evolutionary distances used to infer the tree. In the final dataset, 45,933 positions were available for evolutionary analyses. Phages PsIn, PsCh, Ps25, Ps12on-D, Ps9, and Ps26S are indicated in red. Phages Ps9 and Ps26S are similar to phages Ps25 and Ps12on-D, respectively.

Neighbor-joining (NJ) phylogenetic trees were reconstructed using the whole-genome sequencing data of the top 38 BLAST hit sequences (including RefSeq sequences). In this phylogenetic tree (Figure 1), the studied phages also clustered with other *Pakpunavirus* members, such as PAK_P1, PAK_P2, and PAK_P4, active against *P. aeruginosa*, showing a divergence of 0.0124 base substitutions per site with both the sequences, confirming the VIRIDIC analysis.

**Figure 2:**
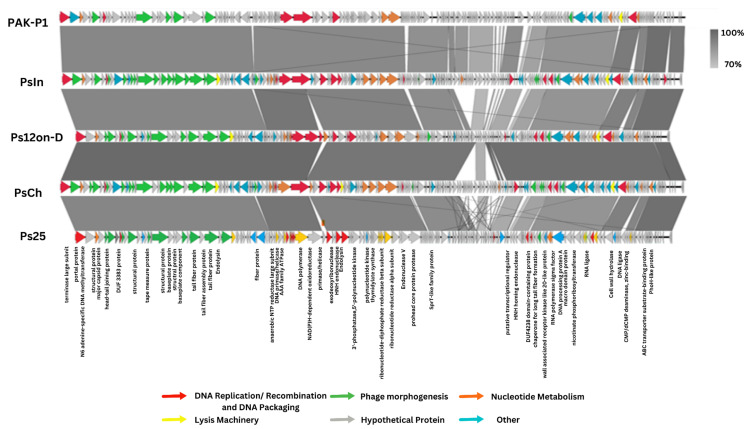
Comparative genome analysis of the *P. aeruginosa* phages PsIn, PsCh, Ps12on-D, Ps25, and PAK_P1. The direction of transcription is shown by arrows next to predicted ORFs. According to the key provided at the bottom of the graphic, arrows are colored according to their functions. The corresponding ORFs are presented with (if available) functional annotations.

A schematic representation of the genomes, with their predicted coding sequence CDSs, functional annotations, and overall genetic organization is shown in Figure 2. Annotation of the phage sequences identified between 164 and 174 coding sequences. Putative functions could be assigned to only about 40 encoded proteins per phage based on sequence similarities (Figure 2). Even though the Ps phages are similar to PAK_P1, we discovered 34 CDSs with no similarities to PAK P1, including 18 genes with no significant similarities to any protein in the database and the rest encoding hypothetical proteins. These are mostly small, ranging in size from 89 to 467 amino acids.

At least 20 genes are suspected to control the nucleotide metabolism system of this set of phages. These genes can direct the host cell’s metabolism into production of progeny phages [52]. They encode enzymes involved in the synthesis of dTTP, such as dCMP deaminase and thymidylate synthase. The latter seems to have diverged from the host bacteria’s precursor [53].

We identified twelve to thirteen tRNAs in the different phage genomes with an average length of 74 bp, which are summarized in Table 1. The presence of tRNA is frequently identified in myoviruses with large genomes [54]. The phage-encoded tRNA genes mostly presented in clusters promote a more rapid overall translation rate and efficiency, especially of rare codons [55]. tRNA deletion has been found to reduce phage fitness [54].

We also observed that all four studied phages encode their own DNA polymerases, which showed high similarity to the DNA polymerases of phages PAK_P1, PAK_P2, PAK_P4 [56], and JG004 [57]. 

Two putative homing endonucleases were detected in the PsCh genome (genes 67 and 87; e-values: 4e-134, 2e-40). The site-specific DNA endonucleases known as homing endonucleases (HEs) are encoded by genes inside mobile elements such as self-splicing introns and inteins (intervening sequences that are spliced and excised post-translationally). Because of their self-splicing activity at the RNA or protein levels, the mobile elements can insert those elements within conserved genes without altering their function [57]. Endonucleases may be involved in the DNA packaging process or in the damage of host nucleic acids. They go through an infection cycle that begins with population invasion, continues with individual spread, and ends when the element is fixed and no longer subject to positive selection. The HE gene sequence degenerates and loses function at this point due to random processes [56]. Surprisingly, phage PAK-P1 lacks a homologue for the putative endonuclease gene 67 found in the genome of the studied phages. One of the studied phages harbored a putative methyltransferase gene (gene 3 in PsIn; e-value: 6e-109). Methyltransferases are important for the methylation of DNA, to protect the DNA against its own endonucleases or endonucleases of the host, which serve as a protection against foreign DNA and infection of phages.

Genes associated with phage morphogenesis also cluster together, among which 17 genes that were predicted to encode structural proteins. The phages are also predicted to produce two phage lytic enzymes: a cell wall hydrolase and an endolysin [47,58]. Although endolysin and holin usually constitute a two-component lysis system to release phage progeny from the host cell, no holin homolog was found in the studied phages’ genomes. 

No sequence homologs to proteins annotated as integrase, repressor, or transposase were found, suggesting that these phages are strictly lytic phages, which is consistent with the results of the highly related phage PAK-P1 [49]. 

The studied phages did not carry any known virulence or pathogenicity genes, as determined by VirulenceFinder and PathogenFinder, increasing their chances to be applicable for phage therapy. 

### 3.3. Host Range Analysis

The newly isolated phages were screened against 140 isolates of *P. aeruginosa* isolated from different sources worldwide (Supplementary Table S2). Most of these strains (n = 124) were of clinical origin and exhibited MDR profiles. Isolation sites of the clinical *P. aeruginosa* strains included infected lungs of CF patients, purulent wounds, otitis, urinary tract infections, osteomyelitis, blood stream infections, and others. Three isolates were isolated from drinking and river waters and from the hospital environment. Two strains were isolated from domestic animals: a dog and a horse (Supplementary Table S2). 

**Figure 3:**
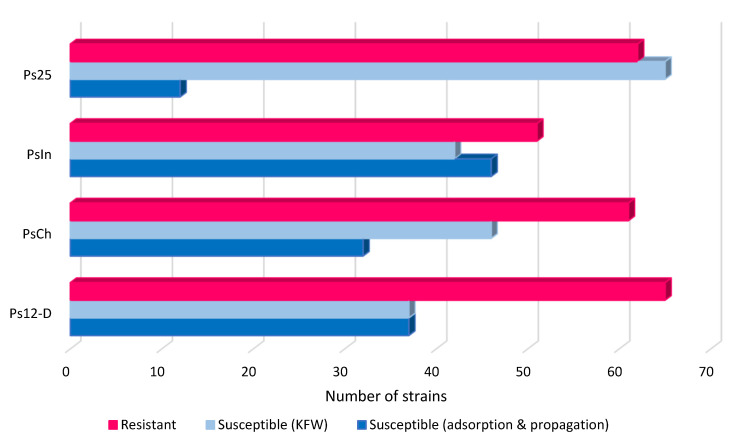
Host range chart of the phages PsIn, PsCh, Ps12on-D, and Ps25, as determined on 140 P. aeruginosa isolates. Resistant: (-) no lysis; susceptible: (KFW) killing from without; susceptible: adsorption and propagation confluent lysis, semi-confluent lysis, opaque lysis, and separate plaque in high and low dilutions.

Overall, Ps12on-D, PsCh, and PsIn showed the broadest host range, capable of productive infections in 37, 32, and 46 out of 140 strains, respectively. The less active phage, Ps25, could infect about 12/140 strains of the collection. However, on many of the bacterial isolates, only killing from without effect (KFW) without propagation within the bacterial cells was observed (Figure 3). According to T Abedon, the phenomenon of lysis-from-without is exhibited by T-even and certain other phages with large genomes, but does not appear to be widespread, and the majority of phages do not cause lysis-from-without; rather, huge multiplicities of infection simply exhaust all potential for effective macromolecular synthesis, resulting in cell death [59]. The most active phage was PsIn, demonstrating a KFW effect on 42/140 strains and successfully infecting 30/140 strains. As the host strain does not contain any bacteriocin-encoding genes, the KFW effect of phages is considered due to the high number of phage particles adsorbing on the tested strains. Whereas the four phages could not infect most strains collected from the environment, phages are capable only of adsorbing on the *P. aeruginosa* strain isolated from drinking water. Regarding the clinical isolates, phages show a productive infection on 53 strains, and most of them were capable of adsorption of all phages, while at least two phages are capable of propagation on 28 strains. Finally, 43 strains were totally resistant towards all four phages (Supplementary Table S2).

Phage PAK_P1, which is similar to the phages in our study, is well-known for its broad host range as well, including *P. aeruginosa* strains recovered from cystic fibrosis patients of primary and chronic colonization cases [49]. In the study by Essoh et al., the phages isolated from Abidjan belonging to the *Pakpunavirus* genus could propagate on 14 out of 28 strains of *P. aeruginosa,* including Pyophage-resistant strains (Pyophage is a commercial phage cocktail from the Eliava Institute (Georgia)) [60]. Furthermore, the high efficacy of the phage cocktail consisting of five phages of the *Pakpunavirus* genus active against a panel of strains, including the well-known laboratory strains *P. aeruginosa* PAO1 and PA1 [39], the mutant strains resistant to the phages PAO1-r-1 and PAO1-w-1, as well as 19 *P. aeruginosa* clinical isolates, was demonstrated by Yang et al [46]. The phage cocktail was able to infect and kill about 90% of the tested strains. Along with eradicating *P. aeruginosa* on solid media, this cocktail was equally effective in liquid cultures and biofilms [46]. 

### 3.4. Thermal and pH Stability

To investigate the effect of different relevant environmental conditions on phage infection, phage stability at various temperatures, pH conditions, and tolerance to storage techniques should also be assessed. To be an appropriate candidate for phage therapy, a phage must be able to withstand different pH conditions and remain infectious for the moment of administration, while tolerating a large variation in pH and temperature during its application [51]. Tolerance to different environmental factors is also an important feature for potential therapeutic phages, as the production process of a number of pharmaceutical formulations implies wide range variability of such factors [61]. 

**Figure 4:**
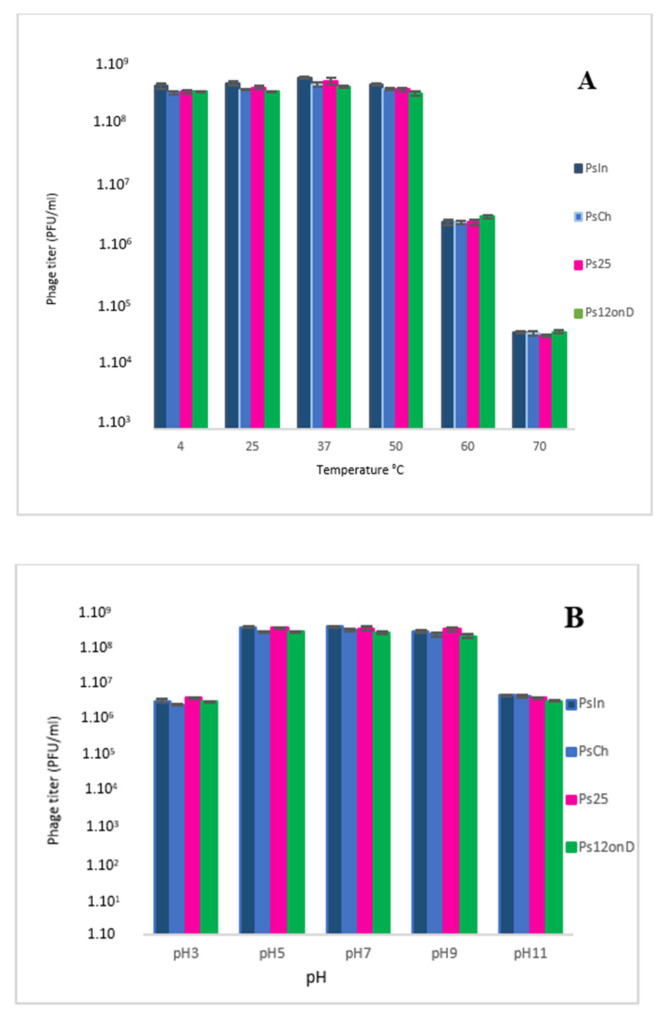
Thermal and pH stability. (**A**) Activity of phages PsIn, PsCh, Ps12on-D, and Ps25 at different pH levels. (**B**) Activity of phages PsIn, PsCh, Ps12on-D, and Ps25 at different temperatures. Each experiment was performed in triplicate and standard deviations are indicated.

The stability of the studied phages was investigated under different thermal conditions. All the phages were stable at approximately 10^8^ PFU/mL after 1 h of incubation at 4 to 50 °C, but their activity decreased sharply with increasing temperature above 50 °C (Figure 4A). The phage titer decreased by a factor of 100 at 60 °C and 1000 at 70 °C.

The stability of the newly isolated phages was also tested at different pH values. The optimal pH level was around 7.0 for all the phages. Reduction in phage titers was observed in both acidic and basic environments. Phage particles remained stable in the pH range of 5–9 (Figure 4B). Upon exposure to pH 3 and pH 11, phage titers dropped below 10^7^ PFU/mL. 

Our data show that the studied phages are quite stable at broad temperature and pH conditions. A previous study demonstrated high stability of phage PA-YS35, another *Pakpunavirus* [52], at the temperature range of 20 to 60 °C and a pH range of 4-9 [52]. Stability of phages at a wider pH range is important for their preservation and clinical use as phage therapy [62].

The infectivity of the studied phages remained stable in a temperature range of 4–50 °C and a pH range of 5–9. 

Thermal stability tests were performed on the phages in order to define their heat-resistant activity. Our phages proved to be temperature-stable, which is an important feature for production process development of a phage therapeutic product in future. However, our data were not much different from previous findings that resulted from experiments, with similar phages showing stability of activity within the same range of pH and temperature conditions [52].

### 3.5. Adsorption Assay 

Adsorption was tested on the four phages and all four of them presented relatively similar curves (Supplementary Figure S2). The infection cycle of the phages was studied on *P. aeruginosa* strain CN573, which was preferred over the isolation strain PaTun, in which a prophage was shown to be induced. This was later confirmed by detection of several prophage regions in the genome of the PaTun strain. The EOP (efficiency of plating) of the tested phages on the strain CN573 compared to PaTun was 1.0. *P. aeruginosa* strain CN573 strain is known as a propagation strain for a large number of therapeutic *P. aeruginosa* phages. Induction of prophages has never been reported in the literature for strain CN573 [22], and the absence of intact prophage regions has been confirmed by a genome analysis performed by Sciensano (the Belgian Scientific Institute of Public Health) to obtain the status of production strains for therapeutic phages, as required in the Belgian monograph for magistral phage preparations [63].

The genome of strain PaTun revealed eight prophage regions in total, ranging from 4.6 to 39.7 kb, including one intact region with a length of 37.9 kb, two questionable regions with lengths of 18.4 and 33.6 kb, and five incomplete regions ranging from 4.6 to 28.2 kb. 

**Figure 5:**
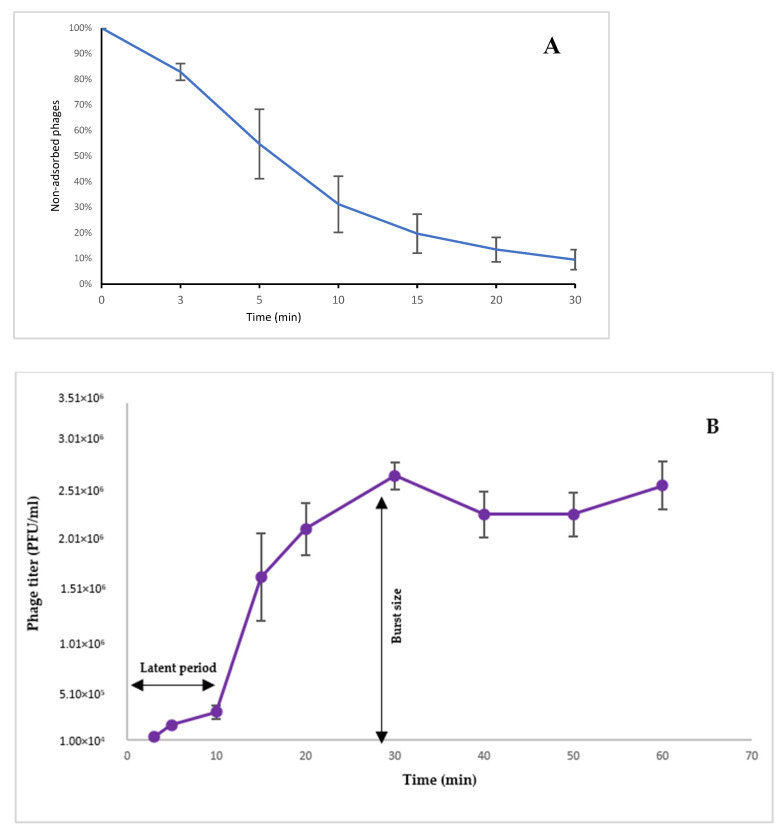
Physiological parameters of phage Ps12on-D. (**A**) The adsorption rate is K = 1.34 × 10^−9^ ml/min of phage Ps12on-D. Phages were mixed with excess *P. aeruginosa* strain CN573, and the non-adsorbed infectious phages were serially counted. Data are percentages of non-adsorbed Ps12on-D relative to the initial input dose of phages. (**B**) One-step growth curve of *P. aeruginosa* phage Ps12on-D on host strain CN573. The latent period is calculated after adsorption. The presented data are means of three independent experiments with error bars showing the standard error of the mean (SEM).

The adsorption experiment for phage Ps12onD on strain CN573 revealed that > 80% of the phage particles adsorbed to the host cells within 10 min (Figure 5A). A previous study showed that for phage vB_PaeM_MAG1, another representative of the *Pakpunavirus* genus, approximately 97% of the MAG1 had adsorbed to the host cells after 10 min [53]. 

### 3.6. One-Step Growth Assay 

As all four studied phages belong to the same species and the adsorption assay showed high similarity, the one-step growth experiment was decided to be conducted on one representative phage: Ps12-D. The experiment was performed on host strain CN573, to determine the latent period and phage burst size. As shown in Figure 5B, the latent period for Ps12on-D, the time interval between phage adsorption, and the start of the first burst can be estimated as 10 min. The burst size, the number of phages produced per infected bacterium, was approximately 115 PFU/infected cell. The one-step growth study by Jiang et al. [52] revealed that phage PA-YS35, belonging to the same genus and showing a high sequence similarity to phage Ps12on-D, had a latent period of 9 min and an average burst size of 380 PFU/cell. Another representative of the *Pakpunavirus* genus, phage PaP1, had a latent period of about 20 min, and the average number of PaP1 progeny produced from one host bacterium was about 65 [55]. 

## 4. Conclusions

In this study, 16 *P. aeruginosa* phages were isolated in Tunisia and four of them were characterized as strictly lytic, free of known genetic determinants coding for toxins or antibiotic resistance, and active against a considerably broad range of clinical *P. aeruginosa* strains isolated in different countries, including some renowned MDR strains. Genome analysis revealed that all four phages belonging to the *Pakpunavirus* genus do not appear to encode known virulence- or lysogeny-associated genes. They were shown to be active over broad temperature and pH ranges and one of them, phage Ps12onD, displayed a relatively short latent period and a high burst size. The characteristics presented by these phages indicate that they may serve as good candidates for therapeutic applications. These are, to our knowledge, the first *P. aeruginosa* phages isolated in Tunisia. Further studies should be performed in order to confirm their efficacy in vivo.

## Figures and Tables

**Table 1:** General features of PsIn, PsCh, Ps12on-D, and Ps25 phage genomes.

Feature	Genome PsIn	Genome PsCh	Genome Ps12onD	Genome Ps25
**Genome size**	92,515 bp	92,710 bp	88,705 bp	87,887 bp
**G+C content** **(G+C content host)**	49.28 % (66.17%)	49.30 % (66.17%)	49.14 % (66.17%)	49.13 % (66.17%)
**No. of predicted CDSs**	174	167	166	164
**Predicted tRNAs**	tRNAGln; tRNATrp; tRNAArg; tRNALeu; tRNAIIe; tRNAAsp; tRNACys; tRNAAsn; tRNAPro; tRNAGly; tRNAPhe; tRNAGlu	tRNAGln; tRNATrp; tRNAArg; tRNALys; tRNALeu; tRNAIIe; tRNAAsp; tRNACys; tRNAAsn; tRNAPro; tRNAGly; tRNAPhe; tRNAGlu	tRNAGln; tRNATrp; tRNAArg; tRNALeu; tRNAIIe; tRNAAsp; tRNACys; tRNAAsn; tRNAPro; tRNAGly; tRNAPhe; tRNAGlu	tRNAGln; tRNATrp; tRNAArg; tRNALeu; tRNAIIe; tRNAAsp; tRNACys; tRNAAsn; tRNAPro; tRNAGly; tRNAPhe; tRNAGlu

## Data Availability

The *Pseudomonas* phage genome sequences were deposited in NCBI. The accession numbers are OM870967 (Ps12on-D), OM870968 (Ps25), OM870969 (PsCh) and OM870970 (PsIn).

## Supplementary Materials

The following supporting information can be downloaded at: https://www.mdpi.com/article/10.3390/v14112339/s1, Figure S1: VIDIRIC-calculated percentage sequence similarity between phages. The phage names are indicated by the horizontal and vertical coordinates.; Figure S2: The adsorption curve of phages PsIn, PsCh and Ps25 on *P. aeruginosa* strain CN 573.
