# Supplementary material for: Isolation and Characterization of Lytic Pseudomonas aeruginosa Bacteriophages Isolated from Sewage Samples from Tunisia
Source: Viruses. 2022 Oct 25;14(11):2339. doi: 10.3390/v14112339 (PMC9698164; doi:10.3390/v14112339)
Supplement: Supplementary file 1 [file viruses-14-02339-s001.zip › viruses-1953580-supplementary.pdf]

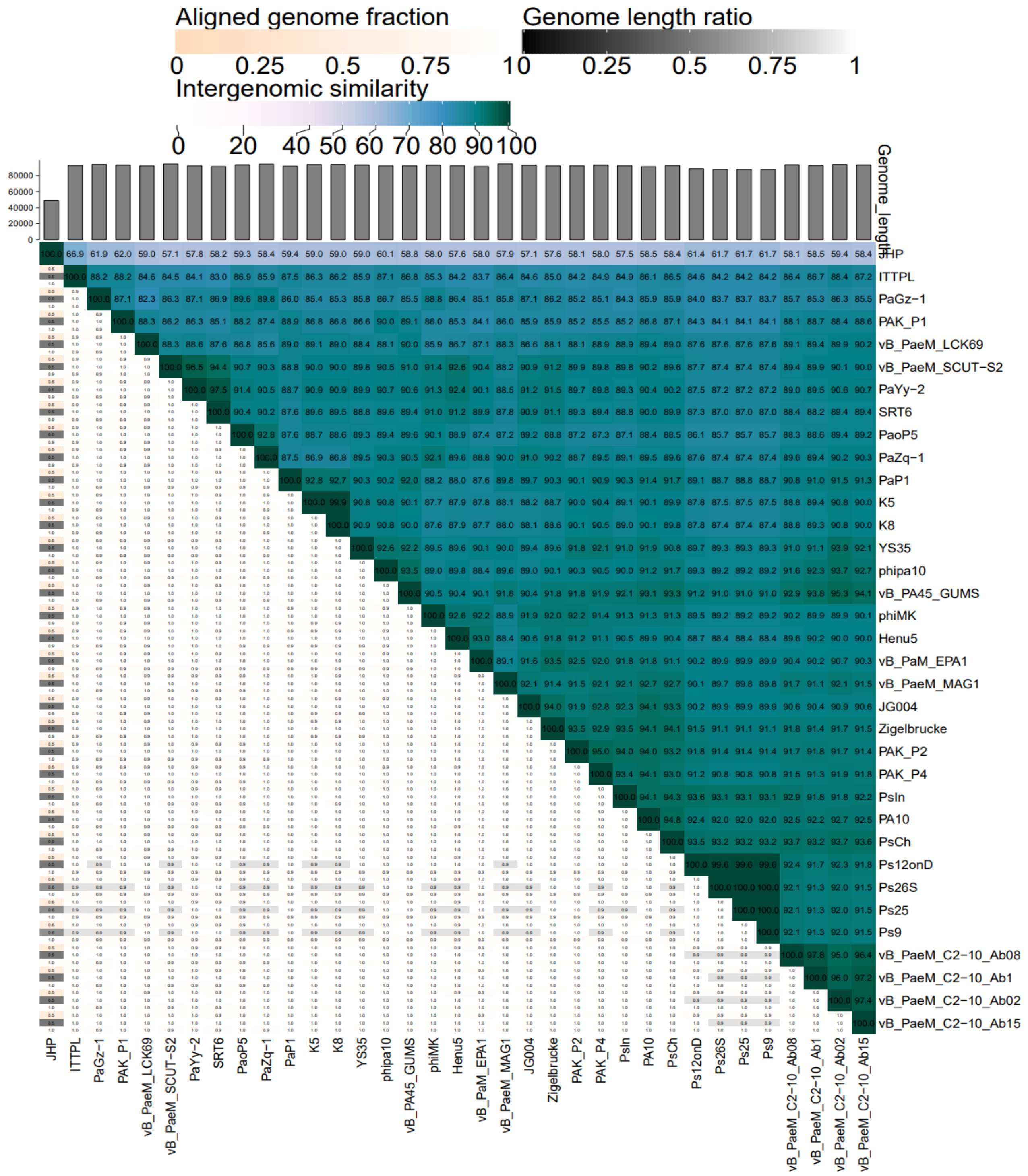

Supplement Figure S1: VIDIRIC-calculated percentage sequence similarity between phages. The phage names are indicated by the horizontal and vertical coordinates.

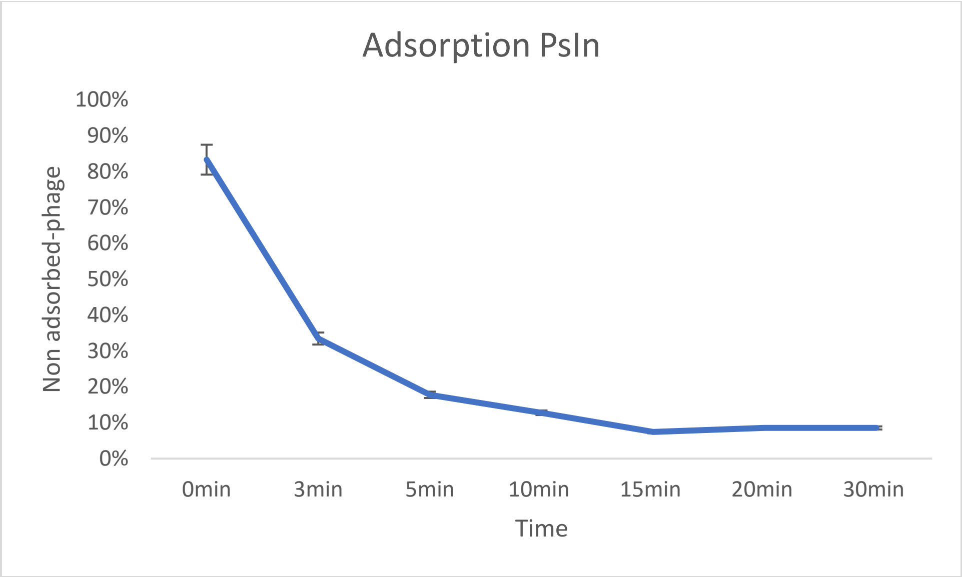
Adsorption PsIn
100%
90%
80%
70%
60%
50%
40%
30%
20%
10%
0%
Non adsorbed-phage
0min
3min
5min
10min
15min
20min
30min
Time

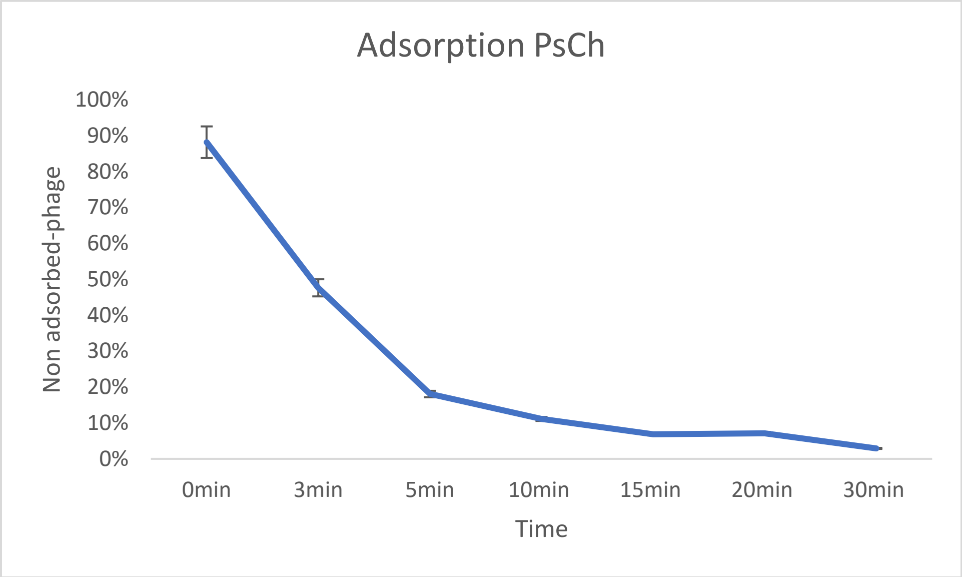
Adsorption PsCh
100%
90%
80%
70%
60%
50%
40%
30%
20%
10%
0%
Non adsorbed-phage
0min
3min
5min
10min
15min
20min
30min
Time

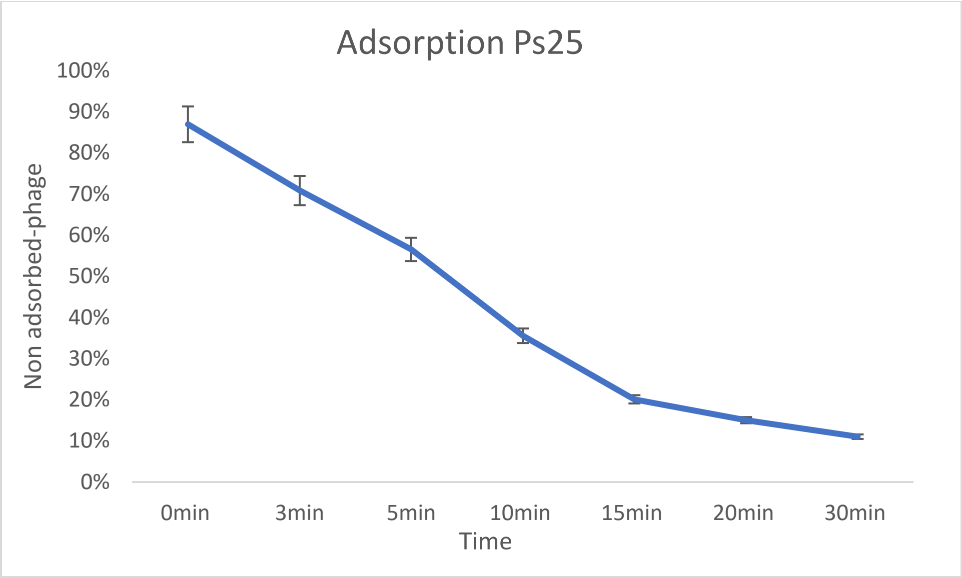


Supplement Figure S2: The adsorption curve of phages PsIn, PsCh and Ps25 on *P. aeruginosa* strain CN 573.
